# Towards Cell-Permeable Hepatitis B Virus Core Protein Variants as Potential Antiviral Agents

**DOI:** 10.3390/microorganisms12091776

**Published:** 2024-08-28

**Authors:** Sanaa Bendahmane, Marie Follo, Fuming Zhang, Robert J. Linhardt

**Affiliations:** 1 Private Faculty of Health Professions and Technologies, Private University of Marrakech, Marrakech 42312, Morocco; 2 Department of Medicine I, Medical Center—University of Freiburg, Faculty of Medicine, University of Freiburg, 79085 Freiburg, Germany; marie.follo@uniklinik-freiburg.de; 3 Department of Chemical and Biological Engineering, Rensselaer Polytechnic Institute, Troy, NY 12180, USA; zhangf2@rpi.edu (F.Z.); linhar@rpi.edu (R.J.L.)

**Keywords:** protein transduction domain, cell-penetrating peptide, HBV core protein, cellular uptake mechanism, heparan sulfate proteoglycan, drug delivery

## Abstract

Hepatitis B virus (HBV) infection remains a major health threat with limited treatment options. One of various new antiviral strategies is based on a fusion of *Staphylococcus aureus* nuclease (SN) with the capsid-forming HBV core protein (HBc), termed coreSN. Through co-assembly with wild-type HBc-subunits, the fusion protein is incorporated into HBV nucleocapsids, targeting the nuclease to the encapsidated viral genome. However, coreSN expression was based on transfection of a plasmid vector. Here, we explored whether introducing protein transduction domains (PTDs) into a fluorescent coreSN model could confer cell-penetrating properties for direct protein delivery into cells. Four PTDs were inserted into two different positions of the HBc sequence, comprising the amphiphilic translocation motif (TLM) derived from the HBV surface protein PreS2 domain and three basic PTDs derived from the Tat protein of human immunodeficiency virus-1 (HIV-1), namely Tat4, NP, and NS. To directly monitor the interaction with cells, the SN in coreSN was replaced with the green fluorescent protein (GFP). The fusion proteins were expressed in *E. coli*, and binding to and potential uptake by human cells was examined through flow cytometry and fluorescence microscopy. The data indicate PTD-dependent interactions with the cells, with evidence of uptake in particular for the basic PTDs. Uptake was enhanced by a triplicated Simian virus 40 (SV40) large T antigen nuclear localization signal (NLS). Interestingly, the basic C terminal domain of the HBV core protein was found to function as a novel PTD. Hence, further developing cell-permeable viral capsid protein fusions appears worthwhile.

## 1. Introduction

Hepatitis B virus (HBV) is a hepatotropic enveloped virus belonging to the *Hepadnaviridae* [1]. HBV is a major public health problem, with an estimated 254 million people worldwide living with chronic HBV infection in 2022 and 1.2 million new infections each year. Although the disease can efficiently be prevented through vaccination, it is still a significant cause of death [2]. The HBV genome in virions is a partially double-stranded relaxed circular DNA (rcDNA) molecule of about 3.2 kbp in length. It comprises four overlapping open reading frames (ORFs) that code for seven different proteins. These include the large, middle, and small form of the envelope protein, collectively known as hepatitis B surface antigen (HBsAg); the hepatitis B core antigen (HBcAg) and the non-assembling hepatitis B e antigen (HBeAg), a processed and secreted product of the joined precore–core ORF; the multifunctional HBV polymerase; and the regulatory X protein (HBx), which controls HBV transcription from covalently closed circular DNA (cccDNA) [3]. cccDNA is produced from rcDNA in the nuclei of infected cells, and its distinctive stability results in chronic infection that can lead to cirrhosis, liver failure, and hepatocellular carcinoma [4]. Currently, there is no cure for chronic hepatitis B, but antiviral treatment is essential to prevent these complications.

The HBV life cycle begins with entry into the hepatocyte, initiated by binding of the envelope proteins to heparansulfate proteoglycans (HSPGs) but only completed upon a high-affinity interaction of the large envelope protein with sodium taurocholate-cotransporting polypeptide (NTCP) [5,6] and subsequent endocytosis. Loss of the envelope and ensuing maturation of the encapsidated rcDNA enable nuclear transport and disintegration of the nucleocapsid [7] for conversion into cccDNA, the template for transcription of new viral mRNAs. Of these, the greater-than-genome length pregenomic RNA (pgRNA) acts as mRNA for the core protein (HBc) and polymerase; in addition, it is co-encapsidated with the polymerase into newly assembling capsids and reverse-transcribed into rcDNA. Via interaction with the surface proteins, progeny nucleocapsids are released from the cell as enveloped virions. Besides the surface proteins as carriers of virus-neutralizing antibody epitopes, HBc has come into focus as an antiviral target due to its multiple dynamic roles in HBV replication and infection [7]. Some small-molecule capsid-assembly modulators have made it into phase 3 clinical trials, but a curative breakthrough is thus far lacking from this and other approaches.

HBc consists of 183 amino acids (subtype ayw) with a molecular mass of approximately 21 kDa. The HBc sequence is divided into an N-terminal domain (NTD) comprising the first 140 amino acids, which is sufficient to mediate assembly of the icosahedral capsid shell [8,9]. This assembly domain contains between aa 75 and 82 the main immunogenic c/e1 epitope, located at the tip of prominent spikes in the three-dimensional (3D) structure [7,10]. The spikes are formed through the dimerization of two assembly domains, and 120 dimers generate the major class of capsids [7,10]. Separated by a nine-residue linker (aa 141–149) follows the highly basic nucleic-acid-binding C-terminal domain (CTD; aa 150–183), which is rich in arginine. Its nucleic acid binding capacity is modulated by differential phosphorylation of interspersed Ser and Thr residues [11].

Currently, many novel therapies with different mechanisms of action targeting the life cycle of HBV are in clinical trials, including entry inhibitors, small interfering RNAs (siRNA), antisense oligonucleotides (ASO), capsid assembly modulators, nucleic acid polymers (NAPs), and immunomodulators [12]. Thus far, however, none has achieved durable off-treatment suppression of HBV replication, even through the combination of treatments examined so far. Hence, searching for novel alternative strategies is still warranted. CoreSN, specifically targeting the encapsidated viral genome, represents one such alternative strategy. So far, this capsid-targeted viral inactivation approach has been demonstrated in cells co-transfected with expression vectors for HBV as the target plus the therapeutic fusion protein [13]. Directly introducing the fusion protein into the HBV-infected cell via incorporation of a PTD, also known as a cell-penetrating peptide (CPP), could represent an advantageous alternative delivery mechanism with potential relevance for other effector proteins, e.g., base-editing or modifying enzymes. Moreover, successful cell penetration would also indicate whether such fusion proteins, when expressed from a therapy-compatible genetic vector, would be able to spread into neighboring cells and possibly enhance antiviral efficacy. All of this requires reliable information on cell entry.

PTDs or CPPs are 5–30 amino acid peptides that often feature a net positive charge, enabling them to interact with negatively charged components of the cellular plasma membrane. This may be followed by internalization through various mechanisms, including endocytosis and direct translocation across the plasma membrane [14,15]. CPPs have been extensively studied for their potential use as transporters for a wide range of molecules, including peptides, proteins, nucleic acids, and small molecules [16]. The original TAT (transactivating transcriptional activator) peptide, derived from the Tat protein of HIV-1, and penetratin, derived from the *Drosophila melanogaster* homeobox protein [17], are among the most widely used CPPs. CPPs can cause cellular toxicity, highlighting the importance of careful optimization and testing of CPPs and their cargo molecules. Several enzymes were successfully imported into cells through conjugation to CPPs, including β-galactosidase [18,19], nucleases, such as Cre recombinase [20], and superoxide dismutase (SOD), which has been transduced into pancreatic beta cells from a diabetic mouse model to protect them from destruction [19]. While such functional data indicate that at least some cells have been penetrated, more direct demonstration of uptake has often produced controversial results, in particular when indirect immunofluorescence of fixed cells is used for detection, which can be prone to post-fixation artifacts regarding the original location of the protein in question.

One of the mechanisms by which CPPs can interact with cells is by binding to HSPGs [21]. HSPGs are glycoproteins found in the extracellular matrix and on the surface of many cells, including cells of hepatic origin [21] yet also on cells that are widely used in cell biology, such as HeLa cells. HSPGs consist of a protein with covalently bound heparan sulfate chains (HS), long linear polysaccharides composed of repeating disaccharide units. Negatively charged HS chains can interact with positively charged CPPs to promote their binding to and uptake by cells [22]. In this work, our objective was to examine the potential of PTD-fused HBV core proteins to enter cells when added to the outside. While a TLM-conjugated HBV core protein has previously been described [23], we aimed to compare this amphiphilic PTD with three other basic-type PTDs. Furthermore, to avoid any uncertainties related to the use of fixed cells, we chose GFP as fusion partner, allowing for the direct monitoring of fusion protein interactions with live cells.

## 2. Experimental Procedures

### 2.1. Plasmid Constructs

The parental expression vector used in this work was pET28a2 [24] which features resistance to ampicillin, a T7 RNA polymerase promoter, and an efficient ribosome binding site (RBS). For N-terminal PTD fusions, its derivative pET28a2-HBc148-G-ctGFP_H6 was used, which encodes HBc1-148 fused to a C-terminally His_6_-tagged enhanced GFP (eGFP; F64L S65T; [25]) via a linker with the sequence A(G_3_S)_3_G_4_T. For PTD insertions into the c/e1 epitope, plasmid pET28a2-c149eGFP [24] was used, which encodes HBc149 with eGFP flanked by linkers (GTG_4_SG_4_; G_4_SG_4_T) incorporated between HBc aa 78 and 80. The eGFP coding sequence was then replaced by appropriate PTD-encoding PCR products (constructs HBc148-c/e1PTD-G-ctGFPH6). N-terminal PTD-fusions started with the sequence MAH_6_GG followed by the desired PTD sequence, joined through 6 to 7 aa linkers to the starting Met residue of HBc148-G-cfGFPH6 (constructs NH-PTD-HBc148-G-ctGFP-H6) or GFP-H6 (control constructs NH-PTD-GFP-H6). The His_6_ tag preceding the PTD sequence was important to prevent rapid proteolysis. For the fusion of one (NLS1) or three copies (NLS3) of the SV40 large T NLS (PKKKRKV) to the C-terminus of GFP (constructs GFP-NLS1/3-H6) or NH-PTD-GFP-J6 (constructs NH-PTD-GFP-NLS1/3-H6), the respective sequences were joined via 8 aa linkers to the C-terminal K238 residue of GFP, followed by a 4 aa linker and a His_6_ tag.

For fusion of the HBc CTD to GFP, the HBc sequence from aa 146 to 183, flanked by G_4_SGM (upstream) and LEH6 (downstream), was analogously joined to the C terminus of GFP (construct GFP-HBcCterm.H6). All constructs were verified through DNA sequencing.

### 2.2. Expression and Characterization of Fusion Proteins

For expression, the desired plasmid was transformed into *E. coli* BL21(DE3)*Codonplus cells (Stratagene, San Diego, CA, USA). Induction using 100 mM of isopropylthiogalactoside (IPTG), cell lysis, and the generation of cleared lysates were performed essentially as previously described [24]. In brief, induced cultures were shaken at room temperature for at least 6 h, cells were harvested through centrifugation, and the cell pellet was frozen at −20 °C for at least 1 h. Thereafter, the pellet was taken up in TN50 buffer (10 mM of Tris-HCl, 50 mM of NaCl, pH 7.5) containing lysozyme (1 mg/mL) and 0.5% (vol/vol) Triton X-100 and incubated on ice until the solution became viscous. Nucleic acids were digested by adding benzonase (Novagen, Darmstadt, Germany,) (1 μg/mL) and MgCl_2_ (1 mM), followed by incubation on a rotating platform for 15 min. Cells were further broken through sonication (Branson Sonifier B-12, Branson Ultrasonics Corporation, Danbury, CT, USA) at a 55% (level 5.5) setting with 6 pulses for 10 s, each with a 50 s pause. To remove cell debris, the lysate was centrifuged (SS-34 rotor) at 13,000 rpm for 15 min at 4 °C. The His_6_-tagged proteins were then enriched through immobilized metal ion affinity chromatography (IMAC) on Ni^2+^–nitrilotriacetic acid (NTA) agarose (Qiagen, Hilden, Germany) under native conditions using stepwise increased imidazole concentrations (20, 50, 250 mM), as suggested by the manufacturer. Usually, 2 mL gel beds were used for the cleared lysate from 200 mL of induced bacterial culture, and elutions were performed using two bed volumes of the respective elution solutions. SDS PAGE of the elution fractions was performed using 12.5% polyacrylamide gels. Proteins were detected through Coomassie Brilliant Blue R250 staining. Protein concentrations were determined using the Bradford assay (BioRad, Hercules, CA, USA) with known concentrations of bovine serum albumin (BSA) as the standard.

### 2.3. Cell Culture and Transduction Protocol

Owing to their beneficial properties for the intended studies, including their relatively large size, adherent growth in monolayers for microscopy, and easy separation into single-cell suspensions for flow cytometry, we mostly used HeLa cells. Cells were cultured in DMEM medium containing 10% fetal calf serum (FCS), 1% penicillin/streptomycin, and 1% non-essential amino acids (all from Gibco, Billings, MT, USA). For protein transduction, the medium from 80% confluent cells plated in a 24-well plate was discarded. Cells were washed twice with 500 μL of serum-free medium and incubated for 30 min to 1 h with 0.5 to 1 μM of FP diluted in 300 μL of serum-free medium. For microscopy, cells were washed twice with 500 μL of PBS to remove excess fusion-protein-containing medium.

### 2.4. Flow Cytometry

To determine the intensity of intracellular GFP fluorescence, cells were incubated with FP in the same way as above but then treated with 80 μL of trypsin (0.05% in EDTA, Gibco) per well for 2 min at 37 °C. Beyond detaching the cells from the plate, this treatment should also degrade FP that is free and/or loosely bound to the outside of the cells, whereas internalized FP is protected. Detached cells were collected through centrifugation, resuspended in 500 μL of PBS containing 5% FCS, and finally transferred to FACS tubes. For each sample, 10,000 events were analyzed (FACS CALIBUR, Becton-Dickinson, Franklin Lakes, NJ, USA). GFP was excited with an argon ion laser at 488 nm, and GFP emission was detected using the FL1: 530/30 filter. Data were analyzed using the CELL-Quest program (B–D, Franklin Lakes, NJ, USA).

### 2.5. Fluorescence and Confocal Laser Scanning Microscopy

Cellular binding and uptake of FPs was imaged with a photomicroscope equipped with an epifluorescence attachment (Axiovert 35, Zeiss, Oberkochen, Germany). GFP signals were observed using 450–480 nm, FT-510, and LP-520 filters. Images were acquired with a CCD camera (Plates Slider diagnostic instrument, Eppendorf AG, Hamburg, Germany) and analyzed with the software “Spot advanced 4.0.9”.

For confocal microscopy, cells were incubated with FPs in 8-well plates (Nunc Lab-Tek II Chambered*1.5 Coglaze System, Thermo Scientific, Waltham, MA, USA); nuclei were stained with DRAQ5 (Thermo Scientific, Waltham, MA, USA). Images were acquired using a Leica TCS SP2 AOBS confocal microscope (Leica Microsystems, Wetzlar, Germany). The objective used was the 63x water immersion lens HCX PL APO lbd (Leica Microsystems, Wetzlar, Germany). BL 63.0 × 1.2 W. Excitation wavelengths were 488 nm for GFP and 633 nm for DRAQ5. The emission wavelengths were 500–560 nm for the GFP and 650–700 nm for the DRAQ5.

## 3. Results

### 3.1. Expression and Purification of Fluorescent PTD Fusion Proteins

**Figure 1:**
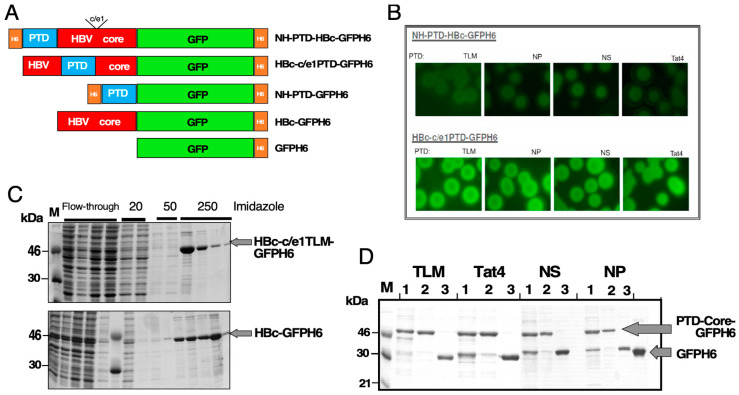
Design, bacterial expression, and purification of fusion proteins. (**A**) Schematic representation of the PTD HBV core constructs showing the insertion of PTDs at different positions within the HBV core protein sequence. PTDs (TLM, Tat_4_, NP, NS) are symbolized by blue boxes; HBc protein sequences by red boxes; GFP by green boxes; and His6 tags by orange boxes. The position of the central c/e1 epitope in HBc is schematically indicated. (**B**) Expression and GFP chromophore formation in transformed *E. coli* bacteria through fluorescence microscopy. *Top row:* N-terminal PTD fusions. *Bottom row:* PTD inserted into the c/e1 epitope. Bacteria transformed with the corresponding expression plasmids were grown on LB agar plates and induced with IPTG, and GFP fluorescence was detected the next day using fluorescence microscopy. (**C**) Enrichment of the FPs by Ni^2+^-NTA IMAC. Lysates from bacteria transformed with the respective constructs were subjected to IMAC. Proteins were eluted by stepwise increases in imidazole concentrations. Equal aliquots from the indicated fractions were separated through SDS-PAGE and detected through Coomassie Blue staining. As representative examples, proteins HBc-c/e1TLM-GFPH_6_ and PTD-less HBc-GFPH_6_ are shown. (**D**) SDS-PAGE analysis of IMAC-enriched proteins. Lanes labeled 1 show the fusions with the indicated PTD inserted into the c/e1 epitope; lanes 2 show the respective fusions to the N-terminus of HBc-GFPH_6_; and lanes 3 show the fusions to the N-terminus of GFPH_6_.

To study the impact of PTD incorporation into HBc on cellular binding and uptake, we generated fluorescent models of HBVcoreSN in which the SN domain was substituted for His-tagged GFP, enabling the direct monitoring of the FP interactions with cells. To this end, we fused four different PTDs either to the N-terminus or into the central c/e1-epitope of the HBc-GFPH6 reporter protein. As PTDs, we opted for the HBV PreS2-derived TLM peptide [26] and for three positively charged HIV-1 Tat-derived sequences, named Tat4 [27], NS [27], and NP [28]. As controls, we generated homologous PTD-GFPH6 fusion proteins without the HBc part; for details, see Table 1 and Figure 1A.

For the development of derivatives of the HBV core protein that feature fused GFP-H6 and PTD peptides at both the N-terminus and within the c/e1 epitope, we used the construct HBc148-G-CtGFP-H6, wherein eGFP is fused to HBc aa 148 via a Gly-rich linker (see Supplemental Figure S1).

In the case of FPs with PTDs at the N-terminus, the respective PTD sequence (TLM, NP, NS, Tat_4_) was joined to aa 1 of HBc148-G-CtGFP-H_6_; an upstream His_6_ tag was essential, as, without it, satisfactory expression in *E. coli* was not achieved. Similar challenges encountered with PTD-GFP-H_6_ (Figure 1A,B,D) were also resolved using an upstream His_6_ tag.

For insertion into the central c/e1 epitope, the PTD sequences were inserted through flexible linkers between aa 78 and 80 of HBc148-G-CtGFP-H_6_ (Figure 1A). Cleared lysates of bacteria transformed with the FP-encoding plasmids were subjected to IMAC on Ni^2+^-NTA columns. After several washing steps with stepwise increasing imidazole concentrations, the desired proteins were eluted with 250 mM of imidazole buffer, as shown through SDS-PAGE of fractions and Coomassie Blue staining (Figure 1A,C). All FPs were well-expressed at levels of 5–10 mg per L of induced culture. Bacterial colonies expressing N-terminal PTD fusions to HBc displayed lower fluorescence inside of *E. coli* cells than PTD insertions into the c/e1 epitope (Figure 1B), independent of the specific PTD. On the other hand, the latter constructs all showed an additional, smaller-than-expected protein band after dialysis (lanes 1 in Figure 1D). The size of about 30 kDa indicates that this smaller product largely comprises GFP, whose structure is highly stable against proteolysis (see also Supplemental Figure S1). The c/e1-inserted PTDs might facilitate intra-HBc cleavage events, leading to degradation of the entire HBc part; however, the mechanisms underlying the appearance of the 30 kDa product were not investigated in further detail as, similarly to the c/e1-PTD constructs, substantial amounts of full-length protein remained, and GFPH6 itself did not detectably interact with cells. We also considered it unlikely that the small amounts of bacterial proteins still present after IMAC would have any marked impact on the GFP-fluorescence-based read-outs. We therefore did not attempt to obtain the FPs in higher purity.

### 3.2. Flow Cytometric Analysis of Cellular Uptake of Fusion Proteins

After successful enrichment of the two sets of FPs in which the PTD was N-terminally added to or centrally inserted into the HBc-part, we first investigated cellular interaction through flow cytometry. Adherent HeLa cells were incubated with the PTD constructs for 1 h and then treated with trypsin for detachment. Trypsin, which hydrolyzes peptide bonds C-terminal of Lys and Arg residues, is also commonly used to remove PTD proteins that adhere only to the outside of cells [29], as confirmed below.

Hence, trypsin treatment prior to flow cytometry allows for distinguishing between cell binding, which is trypsin-sensitive, and cell uptake, whereby the now-intracellular proteins are protected from digestion. Fluorescence signals should thus come primarily from GFP-FPs taken up into the cell, as further substantiated below.

**Figure 2:**
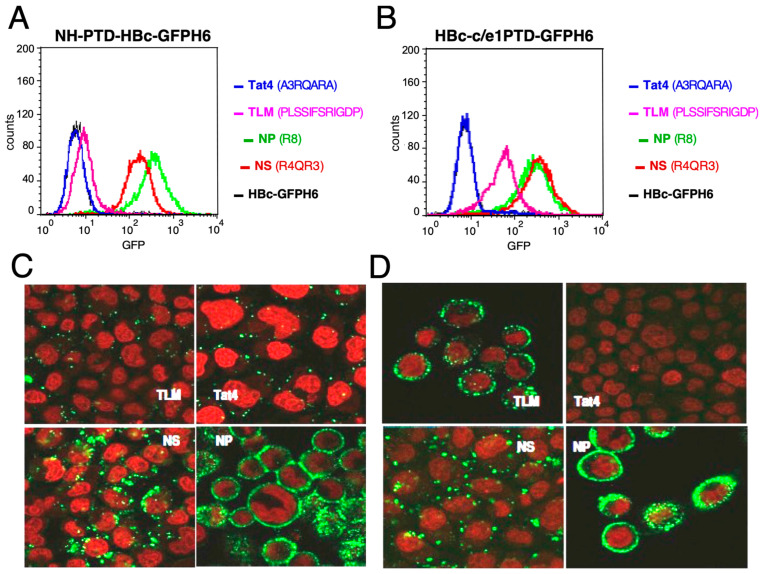
Analysis of cellular uptake of PTD-containing HBc-GFPH6 FPs using flow cytometry and confocal microscopy. (**A**) Flow cytometry profiles of FPs with PTDs at the N-terminus. (**B**) Profiles of FPs with PTD in the c/e1 epitope. HeLa cells were incubated with 1 µM of the indicated FP for 1 h at 37 °C, treated with trypsin for 2 min at 37 °C and two washes with PBS, and analyzed through flow cytometry. (**C**) Confocal microscopy of cells incubated with 1 µM of FP with N-terminal PTD. (**D**) Confocal microscopy of cells incubated with FPs carrying the indicated PTD in the c/e1 epitope. The nuclei were stained with DRAQ5 (red). Note that the scale of individual panels is not always the same; however, an approximate size comparison can be inferred from the Draq5-stained nuclei.

No signals indicating uptake of the constructs containing the Tat4 sequence were detected, as their fluorescence distributions did not differ from the PTD-less HBc-GFPH6 negative control (Figure 2A,B). The construct containing TLM at the N-terminus of HBc-GFPH6 showed only slightly higher fluorescence than the control, while the construct with TLM in the c/e1 epitope gave clearly stronger signals. In contrast, both types of NP and NS constructs showed strong signals, indicating significantly better uptake than the TLM constructs.

### 3.3. Analysis of Cell Uptake Using Confocal Microscopy

To corroborate the intracellular localization of the PTD-containing FPs and to obtain information about their subcellular distribution, we performed confocal laser scanning microscopy of HeLa cells after incubation with the FPs (Figure 2C,D). As in conventional microscopy, cells are observed attached to a solid surface, i.e., without detachment by trypsin; the spatial resolution of confocal microscopy, however, allows for distinguishing binding to the cell surface from a cell-internal localization. The N-terminal PTD FP NH-NP-HBc-GFPH6 showed the strongest fluorescence in the cell periphery, followed by NH-NS-HBc-GFPH6, NH-TLM-HBc-GFPH6, and NH-Tat_4_-HBc-GFPH6, which barely showed a signal. Of the constructs with PTDs inserted into the c/e1-epitope, HBc-c/e1NP-GFPH6 also showed the strongest signals, followed by its NS homolog. HBc-c/e1TLM-GFPH6 also exhibited strong signals, in contrast to the N-terminal TLM fusion. No detectable binding or uptake was seen for HBc-c/e1Tat4-GFPH6. The confocal microscopy data were consistent with flow cytometry results (Figure 2A,B). FPs with NS and TLM showed a punctate pattern indicative of endocytosis, whereas FP with NP showed stronger peripheral staining, but some punctate staining was also detectable. These data indicate that the basic PTDs NS and NP can mediate FP uptake when fused to the N-terminus as well as from within the central c/e1 epitope, whereas the amphiphilic TLM was substantially more efficient inside of the c/e1 epitope. In contrast, the Tat4 peptide showed no detectable uptake activity at either position.

### 3.4. Effect of Insertion of a Nuclear Localization Signal (NLS) Sequence on Cellular Uptake of Fusion Proteins

**Figure 3:**
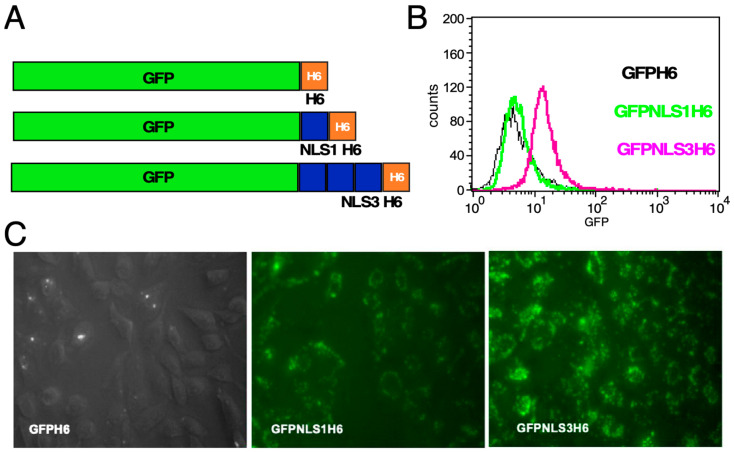
Impact of an NLS on GFPH6 interaction with cell. (**A**) Design of constructs. Dark blue boxes represent the NLS1 and NLS3 sequences, and orange boxes represent the His_6_ tag. (**B**) Flow cytometry analysis of HeLa cells incubated with the indicated GFP proteins. (**C**) Fluorescence microscopy of HeLa cells incubated with the indicated GFP proteins at 1 µM concentration for 1 h at 37 °C and extensive washing. Panel GFPH6 is a merged fluorescence plus bright field image to better visualize cell borders. The few bright spots outside of cells are likely due to precipitated protein.

Conjugation with a nuclear localization signal (NLS) might promote nuclear accumulation of fluorescent FPs that have entered a cell, facilitating detection. A well-known NLS is present in the SV40 large T antigen, with the sequence DPKKKRKV. Single (NLS1) and triple (NLS3) copies of this sequence, as present in commercial vectors, have been observed to induce nuclear import of their fusion partners [30]. On the other hand, the high abundance of basic residues in the NLS strongly resembles the most efficient PTDs, NS and NP. We therefore first investigated the impact of NLS1 and NLS3 sequences on the interaction of PTD-less GFPH_6_ with cells. The respective GFP-NLS derivatives (Figure 3A), and, for the control, the NLS-less GFPH_6_, were first analyzed through flow cytometry of HeLa cells incubated with these proteins. While the single NLS had only a weak effect compared to the GFPH_6_ negative control, the triplicated NLS3 sequence caused a clear fluorescence increase (Figure 3B). Similarly, fluorescence microscopy showed weak cell staining for the NLS1 and much stronger staining for the NLS3 protein (Figure 3C), although this format does not clearly distinguish between binding and uptake.

**Figure 4:**
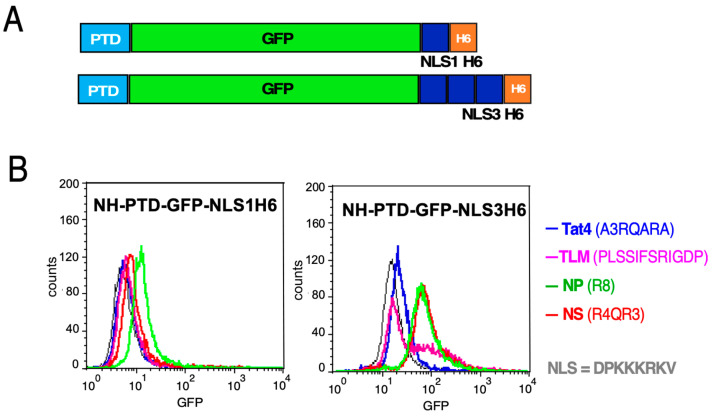
Interaction of NH-PTD-GFPNLS1H6 and NH-PTD-GFPNLS3H6 proteins with HeLa cells. (**A**) Schematic representation of constructs with NLS1 or NLS3. (**B**) Flow cytometry analysis. HeLa cells were incubated with 1 μM of FP for 1 h at 37 °C, washed with PBS, and treated with trypsin for 2 min; trypsin was then inhibited through the addition of FCS. Cells were washed twice with PBS prior to flow cytometry. GFPNLS1H_6_ and GFPNLS3H_6_ (shown in black in the respective panels) were used as controls for the corresponding PTD-containing proteins.

Next, the NLS1 and NLS3 sequences were fused to various NH-PTD-GFPH6 proteins (Figure 4A). Expression was carried out in *E. coli* cells, and the proteins were enriched via their His_6_-tags through IMAC, like the FPs discussed above. Then, HeLa cells were exposed to the FPs and analyzed through flow cytometry, as described above (Figure 4B). In the context of GFP-NLS1-H6, only the NP PTD caused a clear fluorescence increase (Figure 4B, *left panel*), whereas signals for the other PTD FPs hardly changed compared to the PTD-less construct (Figure 4B). In the NLS3 context, both NP and NS led to substantial fluorescence increases, whereas the Tat4 and the TLM sequence had little effect (Figure 4B, *right panel*). The latter result suggests that the impact of the NLS3 sequence is not generally dominant, whereas GFPH6 with the single NLS1 requires a PTD for cellular interaction.

Interestingly, the TLM construct indicated two distinct cell populations, a major one exerting little or no interaction, and a minor one with similar fluorescence intensity, as seen for the respective NP and NS constructs, possibly because TLM mediated uptake or some other kind of protection from trypsin digestion in or on these cells; this was not further investigated. Despite the strong flow cytometry signals with the NP and NS constructs, conventional fluorescence microscopy did not reveal a clear nuclear accumulation for these constructs, perhaps because the FPs remained trapped in endosomes or because the terminally added NLS sequences were intracellularly unstable. Interestingly, the uptake-promoting effect of all tested PTDs appeared weaker for the GFP-only FPs than with the HBc-GFP FPs (see Supplemental Figure S2), indicating a positive contribution of the HBc NTD present in the latter FPs, possibly an adaptation to a PTD-like activity in the full-length HBc protein.

Consequently, the HBV core protein was analyzed for potential PTD activity, particularly the CTD not present in HBc148-G-ctGFPH6 but displaying several NLS domains, as mentioned by [31].

### 3.5. A PTD-like Activity in the HBc-CTD

**Figure 5:**
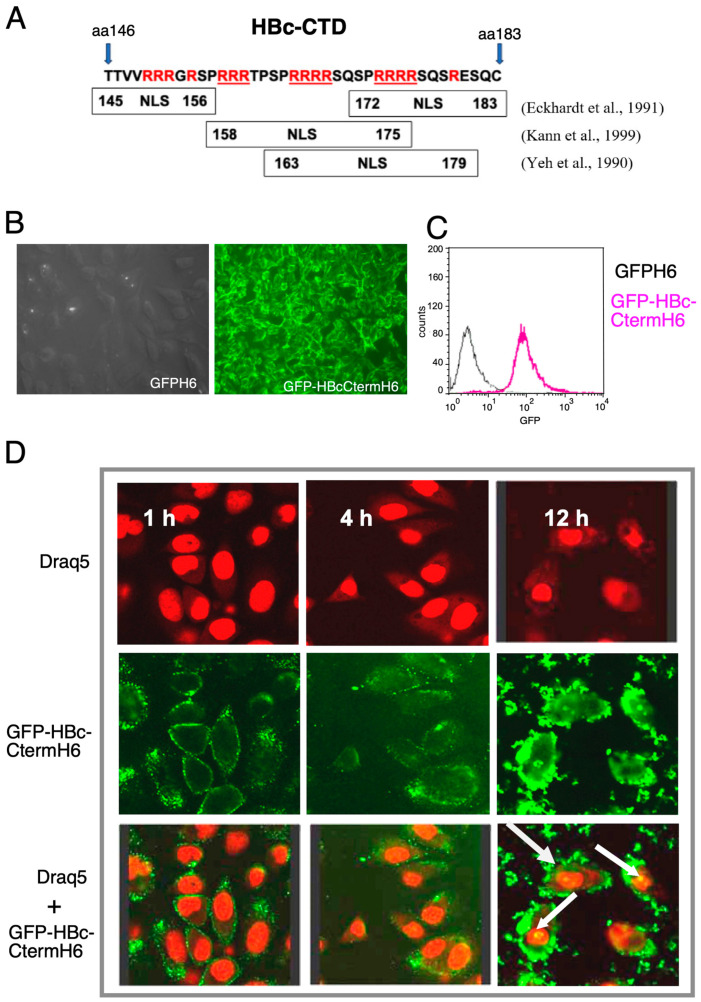
Interaction between GFP-HBc-Cterm.H6 and HeLa cells. (**A**) Amino acid sequence of the HBc CTD. The CTD contains NLSs whose borders have been differently defined; the boxes shown represent those proposed in [31,32,33,34]. (**B**) Fluorescence microscopy of HeLa cells after 1 h of incubation at 37 °C with 1 μM of GFPH6 or GFP-HBc-Cterm.H6. (**C**) Flow cytometry of cells treated with 1 μM of GFPHBcCterm.H6 and GFPH6 as negative control (**D**). Uptake of GFPHBcCterm.H6 by HeLa cells. Cells were incubated with 1 µM of FP for 1 h, 4 h, and 12 h and observed through confocal microscopy. Nuclei were labeled with Draq5 (red). White arrows (*lower right panel*) point to nuclear GFP signals in the 12 h incubation sample, indicating the nuclear localization of some of the FP.

To explore a potential PTD-like activity in HBc, we focused on the CTD, whose many positively charged Arg residues (Figure 5A) resemble the basic NP and NS PTDs as well as the SV40 T antigen NLS, especially its triplicated form. The major function of the HBc CTD is to bind nucleic acids during replication (initially pgRNA, then its reverse transcription product rcDNA). Nucleic acid binding by the CTD is regulated by phosphorylation of the hydroxy amino acids (Ser, Thr) that are interspersed between the Arg residues [11]; a lowered binding capacity is important for rcDNA uncoating and subsequent nuclear cccDNA formation. However, other additional functions of the CTD are not excluded.

To this end, the HBc CTD sequence from T146 to C183 with a C-terminal His_6_-tag was fused through a Gly-rich linker to the C-terminus of GFP (Figure 5A), and the resulting GFP-HBc-Cterm.H_6_ protein was expressed in *E. coli* cells and enriched through IMAC, as described above. Incubation of HeLa cells with the FP resulted in strong cellular fluorescence signals in flow cytometry and fluorescence microscopy (Figure 5B,C). To further investigate cellular uptake, HeLa cells incubated with the FP for different time periods were analyzed through confocal laser scanning microscopy. After 1 h of incubation, binding of the GFP proteins was detected on the cell surface (Figure 5D). After 4 h, green punctate fluorescence was observed inside of the cells, indicative of endosomes. After 12 h, the GFP signal also became visible in the nucleus (*see arrows*). These data are consistent with endocytosis of the FP followed by endosomal release and nuclear import.

**Figure 6:**
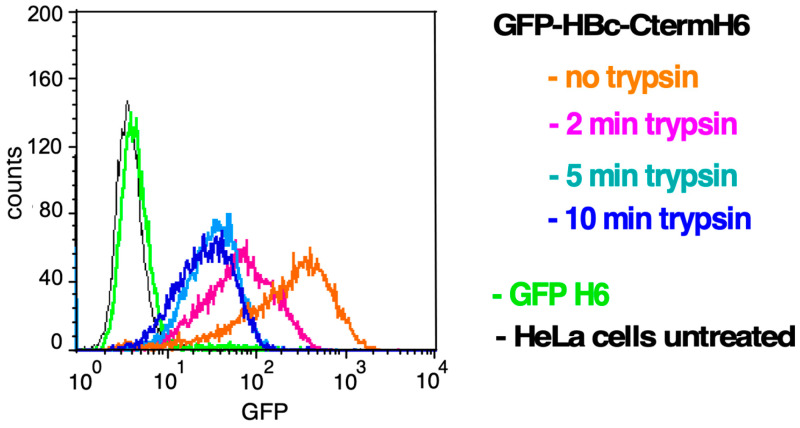
Effect of prolonged trypsin treatment on flow cytometry signals of HeLa cells incubated with GFP-HBc-Cterm.H_6_. HeLa cells were incubated with 1 μM of GFP-HBc-Cterm.H_6_ for 1 h at 37 °C. Subsequently, the cells were treated with trypsin for the indicated time periods prior to analysis through flow cytometry. HeLa cells that were untreated or incubated with GFP-H_6_ served as controls.

To corroborate that the strong cell-associated GFP fluorescence detected through flow cytometry was due to internalization rather than mere cell-surface binding, we compared our trypsin-based cell detachment method with a trypsin-free method. HeLa cells were incubated with GFP-HBc-CtermH_6_ as before and treated with trypsin for different time periods before flow cytometry (Figure 6). Alternatively, cells were removed from the plate with PBS plus 0.02% EDTA instead of trypsin. Cells detached in this way showed a very strong GFP fluorescence intensity. In contrast, 2 min of trypsin treatment (standard conditions) strongly decreased the signals, while treatment for 5 min or 10 min resulted in only a minor further reduction. These data suggest that a short trypsin treatment can remove most cell-surface-bound FP, whereas a substantial fraction of the FP becomes protected by internalization.

### 3.6. Influence of Heparin on the Binding of PTD- and HBc-CTD-Containing FPs to HeLa Cells

**Figure 7:**
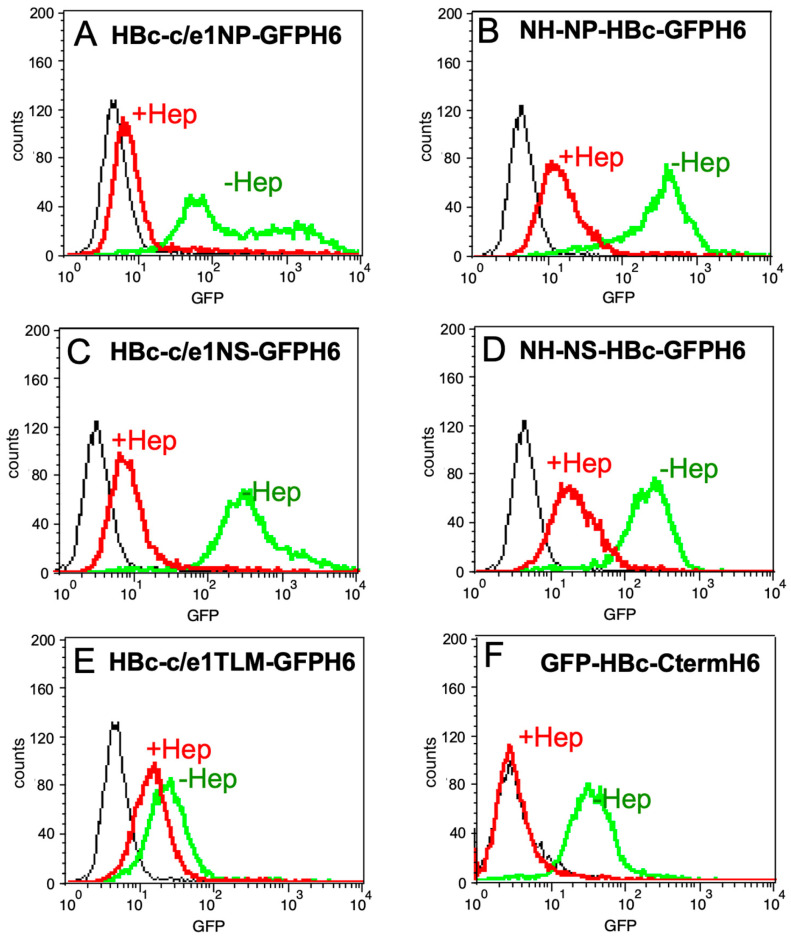
Effect of heparin on the binding of PTD-fusion proteins and GFP-HBc.Cterm-H6 to HeLa cells. HeLa cells were incubated with 1 μM of the following fusion protein (**A**): HBc-c/e1NP-GFPH6, (**B**): NH-NP-HBc-GFPH6, (**C**): HBc-c/e1NS-GFPH6, (**D**): NH-NS-HBC-GFPH6, (**E**): HBc-c/e1TLM-GFPH6 and (**F**): GFP-HBc-CtermH6 in the absence (green curves) or presence of 50 µg/mL of heparin (red curves) at 37 °C. After 2 washing steps, cells were removed by adding trypsin and analyzed through flow cytometry. The black histograms represent the intensity of GFPH6.

Previous studies have shown that heparin modulates the transcriptional activity of Tat [35] and directly binds Tat [36]. Therefore, the cellular uptake of the HBV FPs could also be due to an interaction with HSPGs on the cell surface. To investigate this, the binding of HBc-GFP FPs to the cell surface was analyzed through flow cytometry in the absence versus the presence of heparin. Soluble heparin would compete with heparan sulfates on the cell surface (Figure 7) and thus reduce cell-associated fluorescence.

Treatment with heparin significantly decreased cell fluorescence from the constructs with NP and NS at the HBc N-terminus and in the c/e1 epitope of HBc-GFPH6 (Figure 7A–D). This finding suggests that binding of these FPs to cell-surface HS plays a key role in uptake. By comparison, HBc-c/e1TLM-GFPH6 (Figure 7E) showed less cell association, which, through heparin, was brought down to a similar level as that of the other proteins. The weaker binding inhibition of heparin is consistent with the fact that TLM is not a basic peptide. In contrast, cell binding of GFP-HBc-Cterm.H6 was nearly completely inhibited by heparin (Figure 7F), indicating that binding was more strongly dependent on HS than that of the conventional PTD-containing FPs. Such a dependence on HS has been observed independently by others [37].

## 4. Discussion

The main findings of our study concern the successful cell import of the HBV core-GFP fusion protein through conjugation with different PTDs. The choice of PTDs and their placement in the HBc sequence proved to be important factors. In addition, comparison between PTD-HBc-GFP and HBc-less PTD-GFP fusions revealed an enhancement of cellular uptake by the HBc part. That this might be an adaptation to an intrinsic PTD activity in full-length HBc is supported by the cell-binding and uptake-promoting activity of the HBc-CTD when fused to GFP. Cell interactions of the various HBc FPs were mediated primarily by binding to HS. Cellular import of the PTD-HBc-GFP fusions into HeLa cells under the incubation conditions used (1 μM FP, 1 h, 37 °C) was clearly observed through flow cytometry combined with trypsin digestion of external proteins and through confocal fluorescence microscopy. The efficiency of uptake depended on the specific PTD and its insertion site, especially for the amphiphilic TLM. In general, the highly basic NP and NS sequences and the HBc-CTD displayed the highest PTD activities, as discussed below.

### 4.1. Fusion Proteins with PTD in the Context of GFPH_6_, GFPNLS1H_6_, and GFPNLS3H_6_

Interestingly, our data did not provide direct evidence for uptake into HeLa cells when TLM as PTD was conjugated to GFP H6. In contrast, for 293 cells, an intracellular localization of a TLM-EGFP-H6 fusion protein was reported upon incubation with 2 μM of protein for 1 h [26]. Also, for TLM-HBx and TLM-HBx-GFP proteins, internalization was described [38], and the TLM sequence apparently improved protein immunization efficiency [39]. Hence, the cell type, the FP concentration, as well as the format and sensitivity of the internalization assay can be important for detecting TLM-mediated cellular uptake. Clearly, however, cell binding and uptake into HeLa cells were more pronounced with the basic PTDs than with TLM.

Conjugating an NLS sequence to a PTD FP can direct that protein into the cell nucleus. The NLS is recognized by importing proteins that mediate transport to and through the nuclear pore complex. Such nuclear homing has been exploited for drug design [40,41]. It has also been noted that basic NLS sequences, due to their positively charged nature, could mediate uptake through the plasma membrane [42,43]. In our study, we added a single (NLS1) or a triplicated (NLS3) SV40 large T NLS to PTD-GFPH6 constructs. For TLM as PTD, the addition of NLS3, but not NLS1, substantially enhanced cellular uptake. For Tat4, no uptake was observed, nor for NLS1 or NLS3. However, NP as PTD showed flow cytometry evidence for uptake already with NLS1, and more so with NLS3 (Figure 4). While these data indicate that more positive charges are beneficial for cellular interaction, the effects of PTD and NLS sequences on protein do not appear to be simply additive, and they are also influenced by the specific cargo protein. Given our evidence for a PTD-like function of the HBV core protein CTD, the use of heterologous NLSs may offer no advantages over using full-length HBc for such fusions.

### 4.2. Fusion Proteins with PTD in the Context of HBc-GFPH6

PTD-HBc-GFPH6 fusions displayed, overall, a more efficient interaction with HeLa cells than the GFP-only fusions; however, different PTDs differed in efficiency. Regarding TLM, the constructs with TLM in the c/e1 epitope of HBc-GFPH6 (Figure 2D) showed greater uptake than those with TLM at the N terminus (Figure 2C), in agreement with the flow cytometry results (Figure 2A,B). These differences in uptake likely relate to steric effects. The weak or absent PTD activity of the N-terminal TLM fusion to HBc-GFPH6 seems to be in contrast to the uptake into Huh7 cells and primary human hepatocytes that others have reported for TLM-HBc fusions [23]. However, several experimental differences need to be considered for such a comparison. Besides the different cell types [23], these authors used a complex fusion in which one TLM peptide plus a second, inverted copy were fused as part of a 55 aa extension on the N-terminus of full-length HBc containing the CTD. This construct, expressed in insect cells, formed regular capsid-like particles, which appeared to be translocated in intact form across the plasma membrane. Our constructs carried a single TLM sequence, and the bulky GFP moiety at the end of the HBc NTD prevents assembly into particles [44]; however, GFP as a fusion partner enabled direct rather than indirect, antibody-dependent fluorescence of fixed cell detection, minimizing the risk of post-fixation artifacts. Moreover, although we did not attempt to thoroughly quantitate uptake efficiencies, the higher translocation activity of the basic PTDs compared to TLM in our study is evident.

The least basic of the HIV-1 Tat-derived peptides investigated in our study was Tat4 (A_3_RQARA) featuring only two Arg residues plus one Gln and five Ala residues; the Ala residues had been chosen based on the highest α-helix stabilization value (−0.77 kcal/mol) among the 20 natural amino acids [27]. FITC-labeled Tat4 peptide was reportedly able to transduce Jurka T cells upon incubation at 37 °C for 30 min. However, beyond lacking a protein-sized cargo, flow cytometry detection of uptake was performed without prior trypsin treatment, and the confocal images were obtained using cells fixed with 4% paraformaldehyde, both of which may affect interpretation of these results. Hence, these data are of limited comparative value for our study.

In contrast, our results clearly demonstrate an efficient PTD activity of the highly basic peptides NP and NS, mediating the cellular uptake of the HBc-GFPH6 as well as GFPH6-only fusions. Mechanistically, the strong inhibition of cell binding and an uptake by heparin (Figure 7) indicate that the major initial cell interaction occurs through electrostatic binding to HSPGs on the cell surface, similarly to what has been shown for the original Tat peptide [42,43]. Notably, HSPG binding also represents an important part of HBV infection of hepatic cells, supporting the relevance of our HeLa-cell-based data for HBV as a therapeutic target. Clearly, however, this notion will have to be experimentally corroborated in hepatocytes or hepatocyte-derived cells.

### 4.3. The HBc CTD as a Potential PTD

The higher uptake efficiency of PTD-HBc-GFP versus PTD-GFP fusions suggested a positive contribution of the HBc part, possibly implying an adaptation of the N-terminal HBc assembly domain to an intrinsic PTD activity in the CTD with its many positively charged Arg-resides. Indeed, fusion of the CTD to GFP resulted in strong cellular staining and uptake (Figure 5), which was nearly completely inhibited by heparin (Figure 7). This finding indicates that the HBc CTD mediates cellular uptake through the electrostatic interaction of its basic residues with HSPGs. The respective data from flow cytometry and fluorescence microscopy correlated very well with each other (Figure 5 and Figure 7). In particular, the time course experiments (Figure 5D) provided evidence for entry and likely nuclear import of the GFP-HBc-Cterm.H_6_ protein which, after 12 h of incubation, produced marked perinuclear staining and also apparently intranuclear spots. Together, these data suggest a slow uptake process via endocytosis, followed by endosomal escape and nuclear import of a fraction of the fusion protein. CTD-mediated binding to cell-surface HS has also been independently proposed by others [37]. Their experiments involved intact recombinant capsid-like particles from *E. coli* or *S. cerevisiae.* A role of the CTD in cell binding was therefore surprising, as the CTD in such particles is typically commonly found in the particle’s interior [7]. Our data with a non-assembling CTD fusion protein and the strong inhibition of its cell interaction by heparin confirm the proposed CTD activity, and they suggest that in the cited study at least some of the CTDs were exposed on the particle surface or were present in not fully assembled particles. During HBV infection, destabilization of the nucleocapsid with CTD exposure is likely an integral step of rcDNA-uncoating for nuclear cccDNA formation [7]. To further corroborate and to quantitate binding of the HBc CTD and the basic Tat-derived PTDs to HSPGs, we performed in vitro binding experiments using Surface Plasmon Resonance (SPR) analysis (Supplemental Figure S3 and Supplemental Table S1). Based on the SPR results, no binding was seen by GFPH6, corroborating its suitability as a negative control in our previous experiments. No HS binding was detected for FPs containing TLM or Tat4 in the context of GFPH6, consistent with flow cytometry analyses (Supplemental Figure S2). Again, in agreement with the data described above, GFPNLS3H6 showed a much stronger affinity to HS than GFPNLS1H6 (465 nM versus 442 μM). The HBc CTD in GFP-HBc-Cterm.H6 exerted even stronger HS binding (373 nM) than NLS3. The overall highest affinity was displayed by NP (77 nM), followed by NS (114 nM). Interestingly, the additional presence of NLS1 reduced NP and NS affinity 6-fold (to 420 nM for NP-GFP-NLS1H6) and 12-fold (to 1.48 µM for NS-GFP-NLS1H6), respectively, in accordance with the poor interaction seen through flow cytometry (Figure 4B). These data support the hypothesis that PTD and NLS activities in one molecule are not necessarily additive but may even be mutually inhibitory. This indicates a strong context dependency, which needs to be experimentally addressed for each individual fusion protein.

## 5. Conclusions

This work shows that the basic PTDs NP and NS conjugated to HBc-GFP protein display strong binding to acidic cell surface components of HeLa cells and likely other human cells, given the wide distribution of HSPGs. Furthermore, these basic sequences were able to mediate cellular uptake of their fused protein cargos, most likely via endocytosis. Similar observations were made for GFP fusion proteins containing a triplicated SV40 large T NLS and for the highly basic HBc CTD. In particular, considering that the molecular mass of GFP with around 30 kDa is substantially larger than that of the 17 kDa *Staphylococcus aureus* nuclease employed in the study [45], cellular uptake of a PTD-modified coreSN protein, or of other therapeutically promising HBc fusions, appears feasible. Hence, following and developing this approach appears warranted. However, several issues remain to be explored, including the release of the respective fusion proteins from endosomes, the exclusion of potential cell toxicity, and interferences of the PTD with the co-assembly of the fusion with a wild-type core protein. A straightforward approach might be the coculture of PTD-coreSN or homologous PTD-HBc proteins with HBV-producing cells, which should indicate cell permeability and potential antiviral effects. This could also serve as a model for addressing the ability of vector-produced HBc fusions to translocate into neighboring cells, possibly enhancing antiviral efficacy. However, even if such follow-up experiments are successful, it is likely that these or related capsid protein fusions on their own would not be able to cure chronic hepatitis B; rather, they may be valuable additions to the anti-HBV armament that could become part of more efficient combination therapies than those explored up to now.

## Figures and Tables

**Table 1:** PTD HBV core fusion proteins.

PTD	NH-PTD-HBc-GFPH_6_	HBc-c/e1 PTD-GFPH_6_	NH-PTD-GFPH_6_
TLM: PLSSIFSRIGDP	NH-TLM-HBc-GFPH_6_	HBc-c/e1 TLM-GFPH_6_	NH-TLM-GFPH_6_
Tat_4_: A_3_RQARA	NH-Tat4-HBc-GFPH_6_	HBc-c/e1Tat4-GFPH_6_	NH-Tat4-GFPH_6_
NS: R_4_QR_3_	NH-NS-HBc-GFPH_6_	HBc-c/e1NS-GFPH_6_	NH-NS-GFPH_6_
NP: R_8_	NH-NP-HBc-GFPH_6_	HBc-c/e1NP-GFPH6	NH-NP-GFPH_6_

The first column indicates the name and the amino acid sequence of the respective PTD; the other columns show the domains of the FPs and their relative arrangements.

## Data Availability

The original contributions presented in the study are included in the article and Supplementary Materials, further inquiries can be directed to the corresponding author.

## Supplementary Materials

The following supporting information can be downloaded at: https://www.mdpi.com/article/10.3390/microorganisms12091776/s1, Figure S1: Coomassie Blue gel analysis of GFP fusion proteins after trypsin treatment, Figure S2: Binding analysis of NHPTDGFPH6 constructs to HeLa cells using flow cytometry. Figure S3: Comparison of binding affinities of GFP-HBc-Cterm-H6, GFPNLS3, GFPNLS1H6 and GFPH6 to HS. Table S1: Quantitative comparison of the binding parameters as derived from surface plasmon resonance spectroscopy References [46,47] are cited in the Supplementary Materials.
