# Supplementary material for: Towards Cell-Permeable Hepatitis B Virus Core Protein Variants as Potential Antiviral Agents
Source: Microorganisms. 2024 Aug 28;12(9):1776. doi: 10.3390/microorganisms12091776 (PMC11434381; doi:10.3390/microorganisms12091776)
Supplement: Supplementary file 1 [file microorganisms-12-01776-s001.zip › microorganisms-3077727-supplementary.pdf]

Additional Experimental Results Supporting:

Towards Cell-Permeable Hepatitis B Virus Core Protein Variants as Potential Antiviral Agents

Sanaa Bendahmane [1,*], Marie Follo [2], Fuming Zhang [3] and Robert J. Linhardt [3]

[1] Private Faculty of Health Professions and Technologies, Private University of Marrakech, Marrakech 42312, Morocco
[2] Department of Medicine I, Medical Center—University of Freiburg, Faculty of Medicine, University of Freiburg, 79085 Freiburg, Germany; marie.follo@uniklinik-freiburg.de
[3] Department of Chemical and Biological Engineering, Rensselaer Polytechnic Institute, Troy, NY 12180, USA; zhangf2@rpi.edu (F.Z.); linhar@rpi.edu (R.J.L.)
* Correspondence: s.bendahmane@upm.ac.ma

A. Influence of Trypsin on the stability of fusion protein in a cell-free system:

Fusion proteins underwent initial evaluation for trypsin proteolysis before flow cytometry analysis to gauge stability. The connection between the core protein and GFP proved susceptible to trypsin digestion, attributed to four arginine residues in the linking sequence. To counter this, arginine residues were replaced with a glycine-rich linker lacking basic amino acids, resulting in improved stability compared to those lacking the glycine linker. Consequently, all fusion proteins contained a glycine linker for enhanced stability (Supplemental Figure 1). GFP fusion proteins were incubated with trypsin in a 1:5 molar ratio, with reactions halted at intervals of 10 and 30 minutes using the protease inhibitor PMSF. Protein samples were then analyzed via SDS gel electrophoresis and Coomassie Blue staining.

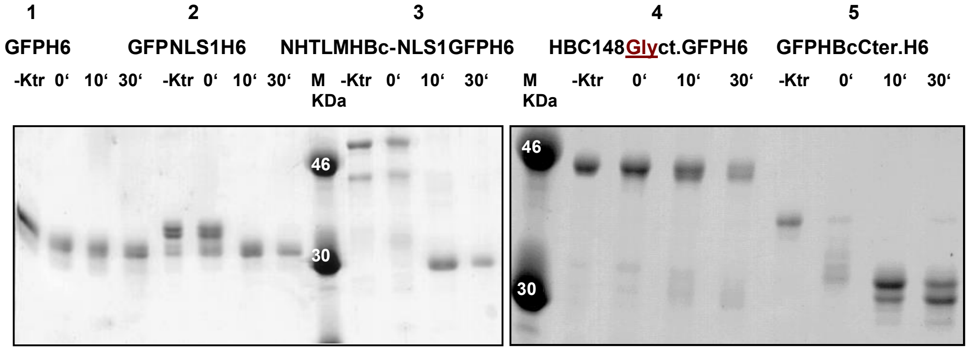

Supplemental Figure S1: Coomassie Blue Gel Analysis of GFP Fusion Proteins after Trypsin Treatment. indicated fusion proteins were incubated with trypsin solution for 0, 10, and 30 minutes. The reactions were halted by adding 1 µl PMSF. Equivalent aliquots were loaded onto a 12.5% SDS gel and detected with Coomassie Blue staining. - Ktr: untreated cells.

GFPH6 was hardly affected by trypsin treatment (Supplemental Figure 1, construct 1). In contrast, construct 2 (GFPNLS1H6), in which a nuclear localization signal (NLS) derived from SV-40 was linked to the C-terminus of GFP, followed by His6, was partially degraded into GFP and other fragments after 10 min of treatment, leaving a product of about 30 kDa. Since the green fluorescence was nevertheless

preserved, it is most likely the GFP from which the additionally fused sequences were split. This also applies to construct 3: NHTLMHBcNLS1GFPH6. Supplemental Figure 1 showed that the original link between core protein and GFP was sensitive to proteolytic digestion (see construct 3). This could be due to the presence of 4-Arg residues in the compound sequence. To test this, the Arg residues were exchanged for a glycine-rich linker sequence without basic AS. These constructs were more stable than those without a gly-linker sequence (see construct 4). Therefore, in the final constructs, the Arg residues were replaced by a glycine linker.

B. BINDING ANALYSIS OF THE PTDGFPH6 FUSION PROTEINS USING FLOW CYTOMETRY

For a comparative investigation of the selected PTDs, HeLa cells were initially incubated with the PTD constructs directly fused to GFP and analyzed by flow cytometry. PTDs with a high number of basic amino acids (NH-NP-GFPH6: 8 Arg and NH-NS-GFPH6: 7 Arg) showed a very slight increase in GFP intensity in flow cytometry (Supplemental Figure 2). However, the weak signals were not detectable under the microscope. The flow cytometry and fluorescence microscopy samples were treated identically, except for the trypsin used to detach the cells for flow cytometry.

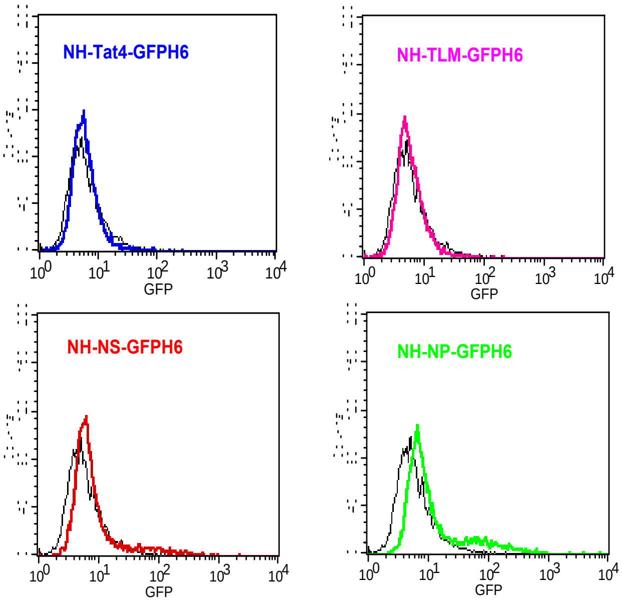

Supplemental Figure S2: Binding analysis of NHPTDGFPH6 constructs to HeLa cells using flow cytometry. HeLa cells were incubated with 1 µM NH-PTD-GFPH6 for 1 hour at 37°C and analyzed by FACS. The black curve represents the GFP fluorescence of GFPH6-treated cells (negative control).

C. Quantitative analysis of binding of GFP fusion protein to HS via Surface Plasmon Resonance (SPR) Spectroscopy.

To study the binding of HBV core proteins to heparan sulfate (HS), surface plasmon resonance spectroscopy (SPR) (Rothenhäusler & Knoll, 1988; Steiner et al., 1999) was performed using a BIAcore3000 (BIAcore, Sweden). Biotinylated bovine liver HS was immobilized on streptavidin (SA)-coated chips according to the manufacturer's instructions. Successful immobilization of HS on the SA coated chips was confirmed by reaching 1000 resonance units (RU). The proteins were stored in a 50% glycerol solution. To remove the glycerol, the proteins were dialyzed against PBS using a dialysis membrane (MWCO 3500). During the SPR experiment, different concentrations of the proteins were added to the surface of the chip at a flow rate of 30 µl/min. After binding of the proteins to HS, the chip was washed with PBS to facilitate dissociation, which took 3 minutes.
For optimal regeneration of the probe layer, the chip was washed with 30 µl of 2 M NaCl followed by 100 mM acetate buffer (pH 4.5). All sensograms were recorded at 25 °C (supplemental Figure 3 and supplemental Table 1). As expected, NH-TLM-GFPH6, NH-Tat4-GFPH6 also showed no binding to HS as both PTDs have only few basic residues. In the context of GFPH6, NH-NP-GFPH6 showed a stronger affinity to HS (KD = 77 nM) as NH-NS GFPH6 (KD = 114 nM). This strong binding correlates with the increased number of Arg residues.

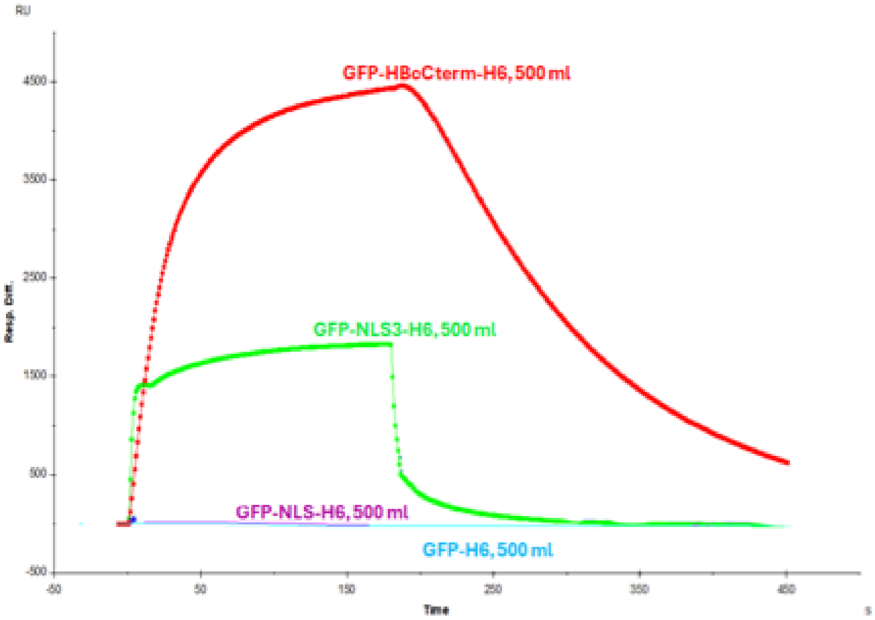

Supplemental Figure S3: Comparison of binding affinities of GFP-HBc-Cterm-H6, GFPNLS3, GFPNLS1H6 and GFPH6 to HS. From top to bottom, SPR sensograms of 500 nM of mentioned proteins are displayed, one above the other. X-axis: RE (resonance unit), Y-axis: time in seconds. GFPH6 alone showed no binding to HS.

Supplemental Table S1: Quantitative comparison of the binding parameters as derived from surface plasmon resonance spectroscopy

	Fusion proteins	K on (1/MS)	K off (1/S)	K_D (M)
	GFP H6	No binding	No binding	No binding
Influence of PTD on the binding	NH-TLM-GFPH6	No binding	No binding	No binding
	NH-Tat4-GFPH6	No binding	No binding	No binding
	NH-NS-GFPH6	$3.98e^{4}$	$1.28e^{-3}$	$1.14e^{-7}$
	NH-NP-GFPH6	$1.14e^{5}$	$8.81e^{-3}$	$7.71e^{-8}$
Influence of PTDNLS$_1$ on the binding	GFP-NLS-H6	132	0.059	$4.42e^{-4}$
	NH-TLM-GFP-NLS H6	1.03e	0.0132	$1.28e^{-3}$
	NH-Tat4-GFP- NLS H6	53.2	0.0176	$3.31e^{-4}$
	NH-NS-GFP- NLS H6	5.11e	0.0756	$1.48e^{-6}$
	NH-NP-GFP- NLS H6	7.84e	0.33	$4.21e^{-7}$
NLS3	GFP-NLS3H6	2.93e	0.136	$4.65e^{-5}$
HBc-Cterm	GFP-HBc-Cterm.H6	3.17e	0.0118	$3.73e^{-5}$

D. SUPPLEMENTAL REFERENCES:

1. Rothenhäusler, B., & Knoll, W. (1988). Surface–plasmon microscopy. *Nature*, *332*(6165), 615-617. https://doi.org/10.1038/332615a0

2. Steiner, G., Sablinskas, V., Hübner, A., Kuhne, Ch., & Salzer, R. (1999). Surface plasmon resonance imaging of microstructured monolayers. *Journal of Molecular Structure*, *509* (1), 265-273. https://doi.org/10.1016/S0022-2860(99)00226-4
